# Testing the effectiveness of a mentoring intervention to improve social participation of adolescents with visual impairments: study protocol for a randomized controlled trial

**DOI:** 10.1186/s13063-015-1028-z

**Published:** 2015-11-05

**Authors:** Eline C. M. Heppe, Sabina Kef, Carlo Schuengel

**Affiliations:** VU University, Section of Clinical Child and Family Studies, Amsterdam, The Netherlands; EMGO Institute of Health and Care Research, VU University Medical Center, Amsterdam, The Netherlands

**Keywords:** Visual impairments, Social participation, Mentor support, Adolescents, Mentoring intervention, Psychosocial functioning, Self-determination, Mentors

## Abstract

**Background:**

Social participation is challenging for people with visual impairments. As a result, on average, social networks are smaller, romantic relationships formed later, educational achievements lower, and career prospects limited. Adolescents on their way towards achieving these goals may benefit from the knowledge and experience of adults who have overcome similar difficulties. Therefore, a mentoring intervention, called Mentor Support, will be set up and studied in which adolescents with visual impairments are matched with successfully social participating adults with and without visual impairments. The main objective of this study is to evaluate the effectiveness of Mentor Support. Secondary aims are to distinguish the importance of the disability-specific experience of mentors, predictors of success, and mediating factors.

**Methods/design:**

The effect of Mentor Support will be tested in a randomized clinical trial, using pre-test one week before starting, post-test after 12 months, and follow-up after 18 months. Participants will be referred to one of the experimental groups or the control group, and this randomization will be stratified according to country region. Three groups are included in the trial: 40 participants will receive Mentor Support by mentors with a visual impairment in combination with care-as-usual, 40 participants will receive Mentor Support by mentors without visual impairments in combination with care-as-usual, and 40 participants will receive care-as-usual only. Mentor Support consists of 12 face-to-face meetings of the mentee with a mentor with an overall time period of one year. On a weekly basis, dyads have contact via email, the Internet, or telephone. The primary outcome measure is improved social participation within three domains (work/school, leisure activities, and social relationships). Mediator variables are psychosocial functioning and self-determination. Predictors such as demographics and personality are also investigated in order to distinguish the pathways to successful social participation. Intention-to-treat and completer analyses will be conducted.

**Discussion:**

The primary outcomes of this trial regard increased social participation. The study may yield insights to further improve effects of support programs to adolescents with visual impairments.

**Trial registration:**

Netherlands Trial Register NTR4768 (registered 4 September 2014).

## Background

Young people with visual impairments (VI) dream about their futures just like everybody else. However, hopes of meaningful relationships with others, exciting leisure activities, successful school careers, and work career pursuits are often dampened due to the difficulties engendered by their disabilities. These difficulties are not only experienced when trying to engage in the activities necessary for successful participation (for example, communication and mobility), but may also result from negative responses from others as well as weaker self-efficacy. Adolescents may therefore benefit when society, including rehabilitation services, supports them in the making of choices, identification of opportunities, and the elimination of obstacles to realizing their goals in social participation [1].

A Dutch longitudinal study of adolescents with VI showed that they have relatively small social networks, fewer friends than their sighted peers, date less, have later sexual debut, have low social competencies, spend more time at home, have less peer activity, have trouble establishing a stable partner relation, and less frequently start a family or have children at a later age. This implies that successful social participation is challenging for Dutch adolescents with VI. Although most of them have a degree and jobs, they had to surmount more obstacles than their non-disabled peers in order to achieve these goals [2–6]. Another Dutch study paints an even bleaker picture for persons with VI regarding employment [7]. Across Europe, persons with VI report less job satisfaction, fewer opportunities, and lower pay [8]. Moreover, research reviews conclude that all over the world persons with VI have distinct challenges regarding social participation and inclusion as well as very low relative employment rates [9, 10].

Building blocks for social participation have been shown to include strong parental, family, and other (such as teacher) support for the participation, a sense of self-efficacy or self-determination, and opportunities to gradually build up experience and skills by “testing the waters” [11–13]. Examples of such gradual stacking include dating as a preparation for steady relationships [14], and having summer or weekend jobs as a preparation for long-term employment [15]. Research indicates that rehabilitation services are still seeking to improve their support for social participation. For example, structured school-to-work programs do not enhance the chances of success in employment [16]. The experiences as studied by McDonnall et al. [15, 16] are also verified by young adults in the Dutch longitudinal study [5]. An important question is therefore: How can factors associated with successful participation be implemented in support? The availability of specific, structured programs, professional support in Dutch rehabilitation centers, or adapted training has not yet proven to be enough.

Mentoring programs may address this need for support. According to Vygotsky [17], a person becomes more competent in interaction with a more competent person. Therefore, jointly accomplishing tasks may lead to better outcomes. This concept of zone of proximal development is consistent with the idea of mentoring, whereby a mentee benefits from a slightly older and more experienced person. Also, Deci and Ryan [18, 19] argue on the basis of the self-determination theory that relatedness is one of the three psychological needs that supports well-being, creativity, persistence, and performance. Relatedness refers to the desire to feel connected, cared for, and loved by others and is a fundamental aspect of mentoring. When supporting adolescents with VI, it is important not to focus only on their anatomic disabilities, but also on the social and environmental context as described with the model of International Classification of Functioning, Disability and Health (ICF) [20]. Mentors interact with mentees within their personal context and, thus, may be well-positioned to address the needs for support for adolescents with VI.

The positive effects of mentoring have been extensively documented [21–27]. Mentoring leads to greater career outcomes, more satisfaction with career outcomes, and more commitment in careers [23, 26]. It also leads to an improvement of quality of life and a lower chance of risk behavior, and it facilitates emotional, social, and psychological growth [24, 25]. One study in the United States of America (USA) [28] found positive results for mentoring adolescents with VI, aged between 16 and 26 years, targeting one domain of social participation (academic achievement and career success). Other mentoring projects for persons with VI in the USA, such as Career Connect and MentorMatch, target only employment. None of these studies or projects aims to achieve participation in all three domains of social participation (employment/school, leisure activities, and social relationships) [9]. In addition, MentorMatch and Career Connect are designed for all vocational ages, are not protocol-based, or are conducted on-line only. Therefore, based on the current state of the field, there was a need to develop and test a more comprehensive mentoring program in the Netherlands.

For the purposes of this research, a mentoring intervention called Mentor Support will be developed and tested for adolescents with visual impairments (mentees), aged between 15 and 22 years. Mentor Support consists of joint meetings with a mentor in or near the mentee’s own (usually home) environment during an overall time period of one year. By performing activities and through modeling, mentees gather information and possible actions and strategies to improve their social participation. Mentor Support is based on existing mentoring interventions in the USA such as MentorMatch, Career Connect, Big Brother and Big Sister, and Home Start and is adapted to our target group and theoretical framework consisting of the self-determination theory, the model of ICF, and the zone of proximal development of Vygotsky. Also, scientific knowledge from a previous national longitudinal study among the target group has been used to develop the program of Mentor Support [2–6].

### Trial objective

In the present study the effectiveness of Mentor Support, a mentoring intervention for adolescents with VI, will be examined by the use of a randomized controlled trial. Participants in one experimental group will receive the intervention from a mentor with a visual impairment, participants in another experimental group from a mentor who is sighted, while participants in the control group will receive only care-as-usual. The main objective of this study is to improve social participation of adolescents with VI. Potential mediating variables, such as indicators of psychosocial functioning and self-determination, and predictors will be investigated in order to distinguish the pathways to successful social participation.

### Hypotheses

The main hypothesis in this study is:

- Adolescents who are assigned to Mentor Support will improve their social participation more strongly in comparison with adolescents assigned to care-as-usual only.

The secondary hypotheses in this study are:

- Adolescents who are assigned to Mentor Support receiving the intervention from a mentor with a visual impairment will improve their social participation as much as adolescents receiving the intervention from a mentor who is sighted.
- Adolescents who are assigned to Mentor Support will improve their psychosocial functioning and feeling of self-determination.
- Adolescents who are assigned to Mentor Support and are self-determined or have a high level of psychosocial functioning will improve their social participation more strongly.

## Methods

### Study design

This study is a superiority randomized controlled trial with multiple arms: two experimental groups and one care-as-usual-only as the control group. Based on equivalence, the two intervention groups receive care-as-usual plus a mentoring intervention called Mentor Support. The difference between these two groups is determined by the VI of the mentors and not by the way Mentor Support is performed. One group receives Mentor Support from a mentor with a visual impairment and one group from a sighted mentor. The effectiveness of this randomized controlled trial will be tested with pre-test, post-test, and follow-up. This study protocol has been approved by the Ethics Committee of the VU University (VCW.1310.010; Netherlands Trial Register NTR4768).

### Study population

Adolescents (*N* = 120) aged between 15 and 22 years with VI are the target population. This study defines visual impairment as “impairment in vision, which even with correction affects an adolescent’s social participation” and, thus, includes both blindness and partial sight. No up-to-date population count of adolescents with VI in the Netherlands exists. Past research estimated the number to be around 1,000 people for an age range of 14 to 25 years [5]. For the seven-year age range used in this study (15 to 22) this could be estimated at 500. The minimum response rate is therefore 24 % if the total population can be reached.

Inclusion criteria are: diagnosed with a visual impairment, age between 15 and 22 years, and living in the Netherlands. Exclusion criteria are: having severe additional impairments, such as complete deafness or intellectual disabilities, and not mastering the Dutch language. For the 120 mentees, 40 mentors will be recruited, half of them having a visual impairment. During Mentor Support mentors will accompany a maximum of two mentees.

### Intervention

Mentor Support is a mentoring intervention developed for adolescents with VI. The primary purpose of Mentor Support is to improve social participation of these adolescents in three domains: 1) work/school, 2) leisure activities, and 3) social relations [9].

In total, Mentor Support consists of 12 face-to-face meetings of the mentee with a mentor within an overall time period of one year. On a weekly basis, dyads also have contact via email, the Internet, or telephone. The joint meetings take place in or near the mentee’s own (usually home) environment and are mostly executed “outside”. Every domain of social participation consists of four meetings, which can be performed in diverse order. Activities accomplished during the meetings are based on mentees’ ambitions and self-designed goals. During Mentor Support mentees gather information, possible actions, and strategies, through modeling, to improve their social participation.

Positive thinking and having success experiences are the main focus of these meetings. Examples of activities can be visiting an (unknown) sports club, gallery, or restaurant, inviting and making friends, and visiting the workplace of the mentor (see Table 1). Mentors and mentees will keep record of their experiences and reflect on their meeting through an evaluation form. All meetings are accompanied by a specially designed systematic handbook consisting of assignments, exercises, and example activities. These tools can also be downloaded by the mentors and the mentees from the Mentor Support website, which is especially designed and accessible for persons with VI. Mentors and mentees have to upload their completed evaluation forms, assignments, and exercises, and, therefore, the website is also used to coordinate the intervention. Modifying the intervention and creating an individualistic path or personalized course is prohibited for several reasons, such as health-related problems within the direct family or moving to another part of the country during Mentor Support.

**Table 1:** Description of themes and domains of each Mentor Support meeting

	Themes of activities	Domain of social participation
Meeting 1	Basic conversation skills first meeting	Social relationships
Meeting 2	Sports and searching for possibilities	Leisure activities
Meeting 3	Timeframe development for the future	School/work
Meeting 4	Life space mapping	Social relationships
Meeting 5	Job visit and searching for vacancies	School/work
Meeting 6	Hobbies and searching for possibilities	Leisure activities
Meeting 7	Preparing a party and inviting new friends	Social relationships
Meeting 8	Presentation skills and appearance	School/work
Meeting 9	Out of the box or out of your comfort zone	Leisure activities
Meeting 10	Searching for your motto in life and role model(s)	School/work
Meeting 11	Discussing romantic relationships, flirting and dating	Social relationships
Meeting 12	Evaluating the intervention during special leisure activity	Leisure activities

Participants randomized to the control group will receive only care-as-usual. The two national organizations for people with VI in the Netherlands offer a wide range of services, such as mobility training, and itinerant teacher support at school. At pre-test, post-test, and follow-up all participants are asked about the kind and amount of care-as-usual service they have received.

### Procedure

#### Mentees

Participants will be recruited through two national service organizations (Bartiméus and Royal Dutch Visio) for people with VI. These organizations have multiple rehabilitation centers, have special education schools all over the Netherlands, and also offer itinerant teacher support to adolescents with VI in regular education. These organizations will send a recruitment brochure and cover letter to inform all clients/students who meet the inclusion criteria and their parents. Clients/students who do not participate will remain anonymous to the researcher. Participants are also to be recruited through online banners, brochures on social media, Internet/magazine/website advertisements, and brochures distributed by several associations for people with VI.

If participants decide to participate, they sign up for this study, with their name and email address, via the website. After signing up, the participants are asked questions about demographic factors, their visual impairment, and possible additional impairments. If participants meet the inclusion and exclusion criteria for this study, they and their parents (if the participant is under 18) receive an official information letter and informed consent form. After signing and sending back the informed consent, they are then eligible to participate in this study. Because signing and sending back the signed informed consent could be challenging for people with VI, participants are allowed to sign the consent form by placing a cross (X) in a box and sending it back by email. Printing and signing by hand is therefore not needed. This procedure has been established with the approval of the Ethics Committee of the VU University.

Once the informed consent is sent back to the researchers, participants receive a baseline measurement (T1). Data will be collected in the Netherlands in their own home environment through computer-assisted telephone interviews. These interviews are conducted by several trained researcher assistants using online survey software called Qualtrics. An interview lasts about one hour and 30 minutes and contains several questionnaires. After this baseline measurement, every participant is randomly allocated to one of the experimental groups or the care-as-usual-only group. The results from the randomization will be sent to the participants in the experimental group (mentees) by email, and the care-as-usual-only group will be informed by telephone.

#### Mentors

All voluntary mentors of Mentor Support will be extensively selected and trained before starting. First of all, the mentors have to sign up through the website, where they enter background information, such as age, impairments, and educational/working achievements. Based on this, eligible mentors will be invited for an intake by telephone. This structured interview contains questions about their own social participation, life experience, and prospects of mentoring. Next, expectations about the intervention will be discussed in order to distinguish eligible mentors. After the intake phase, mentors are invited to attend the Mentor Support mentoring training. Before attending, mentors receive an information letter and have to sign an informed consent. The Mentor Support mentoring training is developed and held at VU University and is partly based on knowledge and experiences from other mentoring programs in and outside the Netherlands. It consists of topics such as: the background and design of the intervention, information about having VI, do’s and don’ts related to etiquette with VI people and safety, information about trust and privacy, and establishing a positive mentor-mentee relationship. During this eight-hour training, mentors have to practice their newly acquired skill by role playing and through assignments about problem scenarios which could occur during the Mentor Support intervention. The training is guided by a train-the-trainer manual. After successfully completing the training, mentors must submit a certificate of good conduct. This document, which must be requested from their own city office, declares that the mentor did not commit any criminal offences that are relevant to the performance of their duties. With this last step, mentors are eligible to accompany mentees. The main objective for mentors, executing the Mentor Support intervention, will be to share life experiences, in order to support their mentee(s). Research has shown that mentors benefit from participating in mentoring programs [29]. Possible positive outcomes could be, for example, an improvement in their social skills, meeting new people, and possibly developing new friendships. After finishing Mentor Support, all mentors receive an official certificate.

#### Matching

**Fig. 1:**
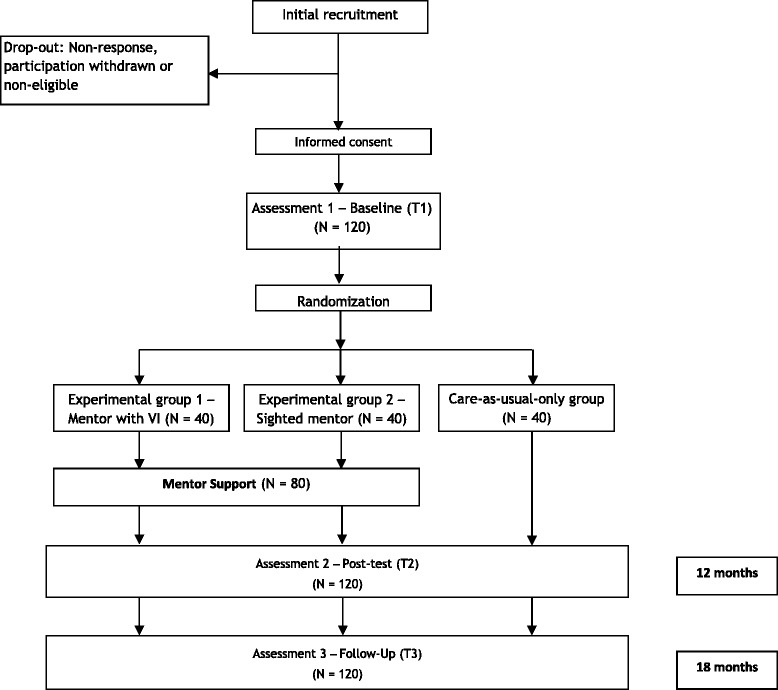
Research procedure

Matching the mentees and mentors is first based on their geographic proximity and subsequently on their similar interests. These interests are measured with a questionnaire. After all mentors and mentees are matched, the primary researcher will notify them by email, and within the same month these dyads begin with the Mentor Support intervention. Directly after Mentor Support has ended, a post-test and a follow-up, after six months, measurement among all participants will be conducted. Figure 1 illustrates the different steps and stages of the research procedure. During the study participants receive information about the study progress through a newsletter. All participants receive a summary report about the effects of Mentor Support at the end of the study.

### Randomization

Upon signing the informed consent, before randomization, participants agree to be allocated to one of the three groups. An independent researcher will randomly assign participants at an individual level to one of the three arms of this randomized controlled study. The allocation will take place with a computerized random number generator after the baseline measurement and before the start of the intervention. Randomization stratification on the important factor of geographic proximity will be used to make sure there is equal allocation to regions. Blocking randomization in each region with a block size of 15 participants will be used to strive for regional groups with the same size. The independent researcher will keep the results of the randomization until the study is completed and all eligible participants are allocated. During the study the primary researcher and participants are not blinded. Only the researchers measuring the post-test and follow-up will be unaware of who is allocated to which arm.

### Measures

#### Primary outcome measures

The main question of this study is whether adolescents with VI improve their social participation after participating in Mentor Support. Several questionnaires will be used to measure social participation within the three domains: work/school, leisure activities, and social relationships.

##### Social participation

An adapted version of the Dutch questionnaire Visuele Activiteiten en Participatie (VAP; Visual Activities and Participation) will be used to measure social participation [30]. Using a 10-point scale, this questionnaire measures the ability to perform a certain task of daily living and the participant’s perception about whether this performance is normal or problematic. The original questionnaire for adolescents consists of ten domains, each containing four questions. Answering these questions allows three subdomains to be distinguished: visual competence (Cronbach’s alpha .94), self-reliance (.83), and participation perception (.91) [30]. In this study five of the ten domains will be used, each with four questions, using two subdomains (self-reliance and participation perception).

##### Work/education

To measure type of education, history of education (special or regular schools), and level of education, three closed-ended questions will be used. For employment, 11 questions measuring type of job, voluntary or unpaid employment hours, travel hours, side jobs, and the maximum working hours per week are asked.

##### Leisure activities

To assess the leisure activities of the adolescents, a Dutch translation of the Degree of Peer Activity (DPAL) questionnaire will be used to measure the amount and frequency of peer contact [31, 32]. This questionnaire consists of five items with a six-point scale with answers ranging from “never” to “every day”. The internal consistency was found to be .66 [31]. All participants will furthermore be asked about their activities during leisure time with 20 questions about sports, hobbies, affiliation with political or religious groups, hours spent on activities during leisure time, and reasons for participating in these activities. Using the Internet, including social media, is one of the most common leisure activities performed by adolescents today [33, 34]. Therefore, this study will include four questions to measure type and frequency of Internet and social media use. Also a Dutch version of The Multidimensional Scale of Facebook Use (MSFU), with ten items and a six-point scale, will be adapted and used to measure social media use [35]. The original scale measured three dimensions of Facebook use: passive Facebook use (Cronbach’s alpha .52), active private Facebook use (.66), and active public Facebook use (.84) [35]. In this study the questionnaire is adapted to general social media use, and a seventh answer on the scale, namely “multiple times per hour”, is added. To measure the perceived online social support on social media, a questionnaire with four items and a five-point scale will be used. These questions are based on the family subscale of the Multidimensional Scale of Perceived Social Support [36]. The internal consistency of this scale was found to be .95 [37].

##### Social relationships

The Social Network Map [5, 6, 38–40] will be used to measure the size and composition of the social network of the adolescents. With this scale each sector or category such as family, extended family (for example, uncle, aunt, niece), friends, and professionals/therapists can be outlined. It measures the amount of people in each sector and the importance of these relationships. Social support will be measured by the personal network list (PNL) with the role-relation method [3, 41]. This seven-item questionnaire measures the social support during leisure activities, problems at school/work, and social relationship problems with answers ranging from 10 “not important” to 100 “really important”. The internal consistency was found to be .76, .79, and .81 [41]. Satisfaction with the social network is measured with two items: “*How satisfied are you with the support you get with practical problems?”* and *“How satisfied are you with the support you get for personal problems?”.* Answers are given on a five-point scale. Developing and maintaining romantic relationships is a common interest during adolescence [14, 42, 43]. To assess this topic, 12 questions will be used, for example, “*Do you have a romantic partner*?”, “*How long has this relationship lasted*?”, and “*Have you ever fallen in love?*”.

#### Measures of mediating variables

##### Well-being

General well-being will be measured with a Dutch translation of the well-being measure developed by Cantrill [44], which asks “*How do you feel in general?*”. The response is given on a ten-point scale, by choosing a number between 1 (feel really bad) and 10 (feel really good). Three items are added regarding well-being in the domains of leisure activities/sport, network of friends, and school/work [3, 5].

##### Self-perception social competence

Perceived social competence will be measured with a Dutch version of Harter’s Self-Perception Profile [45]. In this study four domains (physical appearance, sociability, intimate relationships, and social acceptance) of the questionnaire will be used with each of four items. Each item consists of two opposing statements. Participants have to choose the description that fits them best and then indicate whether this description is true or very true for them. The internal consistencies of these subscales were found to be: .77, .69, .56, and .62 [45].

##### Self-esteem

To measure self-esteem, the Dutch version of the Rosenberg Self-Esteem Scale (RSES) [46] will be administered. This scale comprises 10 items (for example, “*I take a positive attitude towards myself*”). Answers are scored on a four-point Likert scale, with an internal consistency of .85 [47].

##### Loneliness

To assess loneliness, the questionnaire of De Jong-Gierveld will be used [48]. This questionnaire consists of 11 items with answer categories: “yes”, “no”, and “more or less”. With the answers to this questionnaire, two subscales of loneliness can be computed: emotional loneliness and social loneliness with an internal consistency of .81 and .68 [6].

##### Acceptance of the impairment

Acceptance of the impairment will be measured using a subscale of the Nottingham Adjustment Scale (NAS) [49, 50]. This subscale contains nine items. An example of an item is: “*I feel bad when I realize what sighted people can do and what I can’t*”. Answers will be scored on a five-point Likert scale. One positively formulated item is added: “*My visual impairment is part of me, but doesn’t determine what I do or think*”. Internal consistency was found to be .81 [5].

##### Coping

Coping will be measured by the Cognitive Emotion Regulation Questionnaire (CERQ) [51]. This scale consists of 36 items and answers will be scored on a four-point Likert scale. With the answers to these questions, nine subdomains of cognitive coping can be distinguished. The internal consistency of the subdomains ranges from .68 to .83 [51–53].

##### Self-determination

Self-determination will be measured with the Basic Psychological Need Satisfaction and Need Frustration (BPNSF) scale [54]. This 24-item questionnaire with a five-point Likert scale measures the basic psychological needs and need frustration for autonomy, relatedness, and competence. The internal consistency ranges between .64 and .89 [54].

#### Predictor variables

Predictors that might distinguish adolescents who benefit from the interventions include demographic factors and personality.

##### Personality

To measure personality, a Dutch version of the Big Five questionnaire with 30 items and a seven-point answer scale is used [55]. From this scale five different domains or dimensions of personality can be distinguished: extraversion, neuroticism, openness to experience, agreeableness, and conscientiousness. The internal consistency ranges from .68 to .90 [56].

##### Demographic characteristics

15 open-ended and closed questions will be used to collect participants’ demographic information concerning gender, age, nationality, ethnic origin, living situation, and characteristics of their home and family.

#### Control variables

Degree of visual impairment, mobility, and treatment integrity will be measured and used as control variables.

##### Degree of visual impairment

The Functional Vision Scale of Weiner [57] will be used to measure functional vision. This six-item questionnaire with answer categories “yes” and “no” categorized participants in three groups: moderate low vision, severe low vision, and blind. Participants will be categorized as blind if they use Braille, as having severe low vision if they cannot read regular print but do not use Braille, and as having moderate low vision if they can read regular print. Further, questions will be asked about the onset and course of the impairment as well as the medical diagnosis. Seven closed-ended questions will be used to measure additional impairments or chronic diseases and one closed question will measure their perceived level of general health.

##### Mobility

Two questions on level of mobility, independence in traveling and use of aids will be used to measure mobility [5].

##### Treatment integrity

To assess whether the intervention is carried out according to the Mentor Support manual, all dyads have to fill out an evaluation form after every meeting. These forms will show what kind of activity, how long, and which assignments or exercises they have performed.

### Sample size

The total sample size is based on the expected differences in outcome measures between participants assigned to the experimental arm receiving Mentor Support and the care-as-usual-only arm. For testing the significance of the between-subject factor (primary hypothesis) a sample of 120 participants (40 in each condition) is necessary to achieve an adequate power of .85 within a medium effect size (d = .5) between the conditions over time with a significance level of .05. Due to dropout or unexpected organizational aspects the number of participants could drop to as low as 108 to achieve a high power of .80. To answer the research question concerning the influence of potential mediators or predictors on the effectiveness of Mentor Support, several repeated measures within-between interaction analyses will be performed. Based on a significant level of .05 and a power of .85 (*f* = .25), a total sample size of 110 is needed.

### Selective attrition

All participants will be expected to participate in pre-test, post-test, and follow-up during this study. All those who drop out of the study during Mentor Support or from the care-as-usual-only group will be measured for post-test and will be followed up if possible. Dropout reasons will be documented and analyzed in order to use this information for improvement of the execution of the intervention. To account for selective attrition, intention-to-treat analyses will be performed.

### Data analyses

Most analyses will be conducted using the software program Statistical Package for the Social Sciences (SPSS). The descriptive analyses will be carried out using standard methods. If necessary, outliers will be checked and winsorized before analyzing. Differences in baseline characteristics between both experimental groups and the care-as-usual-only group will be analyzed and results will be controlled for these differences if needed. For analyzing the primary (social participation on three domains), mediation (psychosocial functioning and self-determination), and predictor (demographic factors and personality) outcomes, ANOVA repeated measures or multilevel analyses will be used. The primary outcomes of participants in both experimental groups will be compared separately from the care-as-usual-only group. Pre-test, post-test, and follow-up will show the change in quantity and quality of social participation, psychosocial functioning, and self-determination as compared with the care-as-usual-only group, over time. Feasibility of the Mentor Support intervention will be analyzed using descriptive analyses. General mixture modeling with M-plus [58] will be used to examine the impact of the Mentor Support intervention on subgroups. Analyses and reporting of the results will be done according to the CONSORT Statement guidelines.

### Data management and monitoring

Data will be collected using online survey software called Qualtrics. This software makes it easy to transport data to other software programs such as SPSS to analyze data. There is no risk of participants being involved in data management and, therefore, no Data Monitoring Committee has to be established. This study is financially supported by Vereniging Bartimeus Sonneheerdt and ZonMw, the Dutch Organization for Health Research and Development program InZicht and embedded in the EMGO+ Institute for Health and Care Research of the VU University Medical Center. These three organizations will conduct site visits or audits to check the progress of the study throughout the project. The EMGO+ Institute provides an electronic quality assurance handbook to uniform the conduct and safeguard the quality of research within the institute, and this handbook will be consulted during this study.

### Protection of data privacy

All participants in the study will be assigned a participant number. Key lists will be stored separately from the data and will be deleted after final data analyses. Data will be analyzed in a way such that no conclusions can be drawn about individual participants. All data are stored on lockable laptops in lockable cabinets in lockable rooms. All researchers, research assistants, and students working within the project sign a statement in which they declare not to disclose any information about research participants to a third party.

### Publication policy

The results of this study will be published in international journals. To make the results also available for Dutch service providers, professionals, and participants, we plan to publish the results in Dutch journals. Results will also be presented at international scientific conferences, as well as at national conferences within the field of youth care and visual impairments.

### Ethical considerations

The study protocol has been approved by the Ethics Committee of the VU University (VCW.1310.010). Changes within the study procedures will first be proposed to the Ethics Committee.

## Discussion

This study aims to improve social participation of adolescents with VI and compares two experimental groups with one care-as-usual-only group. A secondary objective is to distinguish if psychosocial factors and self-determination mediate this process and if the two experimental groups lead to different social participation outcomes. A strength of this study is the added value of the use of two types of mentors to improve social participation. This might give us more insight into the importance of experiential expertise during mentoring, especially during the transition phase from adolescence to young adulthood. This effect is still uncertain and has never been studied before. Also, the lack of studies about the effectiveness of mentoring interventions to improve social participation makes this study unique. Targeting three domains of social participation is another strength of this study. It provides information about the outcome factors of mentoring and the connectedness of these domains within social participation. A fourth strength of this study is the use of several well-known, frequently used, reliable questionnaires which measure a large number of key variables and factors. This provides the opportunity to look at predictors and mediating effects over time and to compare results from this study with other (international) studies. Another strength of this study is that the target group is a less frequently studied group of persons. Most of the studies concerning people with VI examine developmental problems such as language development, locomotion, and academic achievement. But the total construct of social participation is mostly neglected, especially during the transition phase from adolescence to young adulthood. This study may therefore expand knowledge about social participation development. Finally, a strong aspect of this study is the use of the Internet during the intervention, with a specially designed website. This allows information to be spread easily and all participants to be contacted in the same way. This is especially salient because participants in this study live in several (sub) urban locations throughout the Netherlands, and also because adolescents are likely to be extremely comfortable with web-based interactions and information flow.

One limitation inherent in this study may be the risk of selective attrition during the relatively long period of 18 months for which the participants are connected with the study. During the Mentor Support intervention mentees could drop out of the study for several reasons, such as moving to another part of the Netherlands, parental concerns, or health reasons. Dropout from the care-as-usual-only group may occur directly after the primary researcher has informed them about the results of the randomization and after 12 months with T2. All participants receive and sign an informed consent whereby they are informed in advance about the possibility of ending up in the care-as-usual-only group. This is also mentioned in the first letter or brochure that they receive. This may prevent dropout from the care-as-usual-only group. Another limitation of this study could be the relatively small sample size, due to the small population size. This could lead to problems with making firm conclusions about predictors and mediating study findings. Finally, a limitation could be to find enough mentors in the same region of the country where the participant lives. In this case more mentors are probably needed than the number 40 that we have described above.

In conclusion, this study will gain knowledge about the effectiveness of a mentoring intervention (Mentor Support) for adolescents with visual impairments to improve their social participation. Three conditions are included to test the importance of disability-specific experience. In addition, this study will also give more insight into the pathways to successful social participation, several predictors, and the mediating effects of psychosocial functioning and self-determination.

## Trial status

The study started in December 2012. After being granted permission by the Ethics Committee of the VU University to start including participants, the first research participants were included in September 2014. At this time, data is being collected from the first wave of 58 participants. Currently, recruitment and data collection are still in progress. We expect the main RCT results to be published at the beginning of 2018.

## Abbreviations

BPNSF: Basic Psychological Need Satisfaction and Need Frustration Scale
CERQ: Cognitive Emotion Regulation Questionnaire
DPAL: Degree of Peer Activity List
ICF: International Classification of Functioning, Disability and Health
MSFU: Multidimensional Scale of Facebook Use
NAS: Nottingham Adjustment Scale
PNL: Personal Network List
RCT: Randomized Controlled Trial
RSES: Rosenberg Self-Esteem Scale
SPSS: Statistical Package for the Social Sciences
T1: First assessment
T2: Second assessment
T3: Third assessment
USA: United States of America
VAP: Visual Activities and Participation
VI: Visual Impairments
